# Supplementary material for: Diversity Analysis and Genetic Relationships among Local Brazilian Goat Breeds Using SSR Markers
Source: Animals (Basel). 2020 Oct 10;10(10):1842. doi: 10.3390/ani10101842 (PMC7600759; doi:10.3390/ani10101842)
Supplement: Supplementary file 1 [file animals-10-01842-s001.pdf]

animals

Supplementary Materials

Diversity Analysis and Genetic Relationships among Local Brazilian Goat Breeds Using SSR Markers

Marcos P.C. Menezes, Amparo M. Martinez, Edgard C. Pimenta Filho, Jose Luis Vega-Pla, Juan Vicente Delgado, Janaina K. Gomes Arandas and Laura Leandro da Rocha

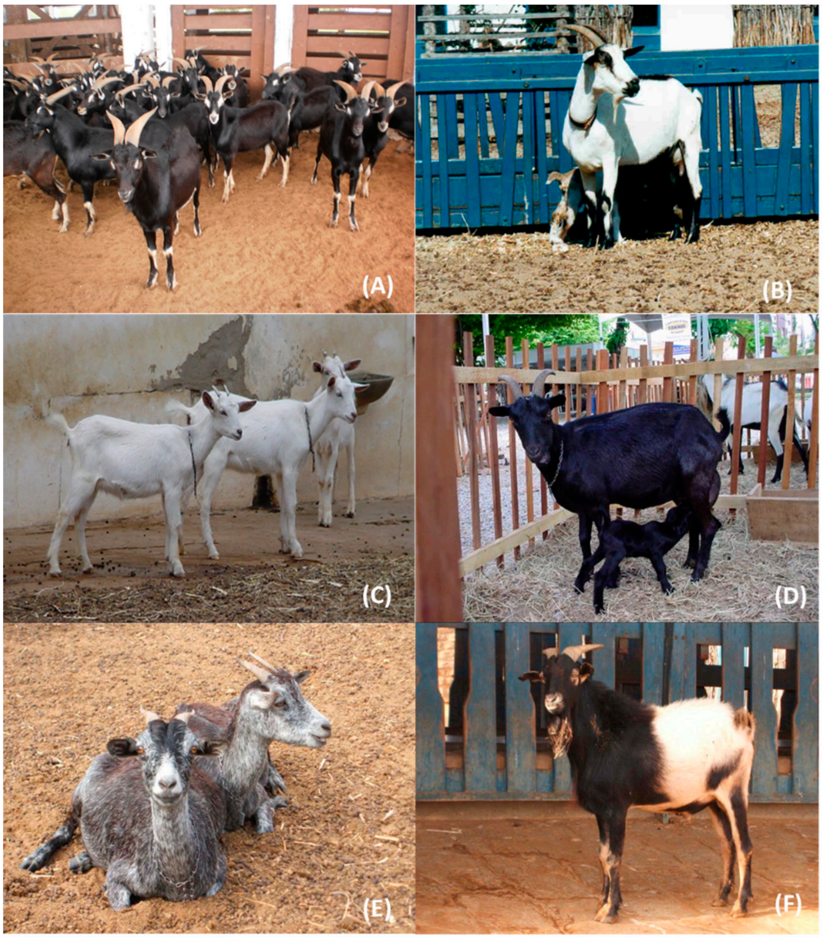

Figure S1. Brazilian goat breeds, (A) = Canindé, (B) = Moxotó, (C) = Marota, (D) = Graúna, (E) Serrana Azul, (E) = Repartida.

Table S1. Microsatellites analyzed, fragment sizes, fluorochromes and the respective sequences (direct and reverse) of the primers.

Locus	**Size**	*Fluorescence*	*Direct*	*Reverse*
BM6506	190–228	HEX	GCACGTGGTAAAGAGATGGC	AGCAACTTGAGCATGGCAC
INRA63	153–185	HEX	ATTTGCACAAGCTAAATCTAACC	AAACCACAGAAATGCTTGGAAG
CSRD247	208–250	FAM	GGACTTGCCAGAACTCTGCAAT	CACTGTGGTTTGTATTAGTCAGG
ETH225	130–165	NED	GATCACCTTGCCACTATTTCCT	ACATGACAGCCAGCTGCTACT
TGLA122	130–180	FAM	CCCTCCTCCAGGTAAATCAGC	AATCACATGGCAAATAAGTACATAC
INRA5	125–155	HEX	TTCAGGCATACCCTACACCACATG	AAATATTAGCCAACTGAAAACTGGG
HAUT27	125–160	NED	TTTTATGTTCATTTTTTGACTGG	AACTGCTGAAATCTCCATCTTA
BM8125	100–128	FAM	CTCTATCTGTGGAAAAGGTGGG	GGGGGTTAGACTTCAACATACG
ILSTS011	253–295	HEX	GCTTGCTACATGGAAAGTGC	CTAAAATGCAGAGCCCTACC
SPS115	235–265	NED	AAAGTGACACAACAGCTTCTCCAG	AACGAGTGTCCTAGTTTGGCTGTG
BM1818	250–290	FAM	AGCTGGGAATATAACCAAAGG	AGTGCTTTCAAGGTCCATGC
CSSM66	175–265	HEX	ACACAAATCCTTTCTGCCAGCTGA	AATTTAATGCACTGAGGAGCTTGG
BM6526	145–195	FAM	CATGCCAAACAATATCCAGC	TGAAGGTAGAGAGCAAGCAGC
INRA6	100–130	HEX	AGGAATATCTGTATCAACCTCAGTC	CTGAGCTGGGGTGGGAGCTATAAATA
MM12	85–135	NED	CAAGACAGGTGTTTCAATCT	ATCGACTCTGGGGATGATGT
OarFCB304	130–180	HEX	CCCTAGGAGCTTTCAATAAAGAATCGG	CGCTGCTGTCAACTGGGTCAGGG
OarFCB11	120–160	FAM	GGCCTGAACTCACAAGTTGATATATCTATCAC	GCAAGCAGGTTCTTTACCACTAGCACC
MAF209	100–125	HEX	GATCACAAAAAGTTGGATACAACGTGG	TCATGCACTTAAGTATGTAGGATGCTG
MAF65	110–152	NED	AAAGGCCAGAGTATGCAATTAGGAG	CCACTCCTCCTGAGAATATAACATG
BM1329	153–185	NED	TTGTTTAGGCAAGTCCAAAGTC	AACACCGCAGCTTCATCC
HSC	270–306	HEX	CTGCCAATGCAGAGACACAAGA	GTCTGTCTCCTGTCTTGTC
McM527	150–190	NED	GTCCATTGCCTCAAATCAATTC	AAACCACTTGACTACTCCCCAA
SRCRSP8	210–260	NED	TGCGGTCTGGTTCTGATTTCAC	CCTGCATGAGAAAGTCGATGCTTAG
INRA23	185–230	HEX	GAGTAGAGCTACAAGATAAACTTC	TAACTACAGGGTGTTAGATGAACTC
CSRM60	75–110	FAM	AAGATGTGATCCAAGAGAGAGGCA	AGGACCAGATCGTGAAAGGCATAG
ETH10	200–230	FAM	GTTCAGGACTGGCCCTGCTAACA	CCTCCAGCCCACTTTCTCTTC
OarFCB48	140–170	FAM	GAGTTAGTACAAGGATGACAAGAGGCAC	GACTCTAGAGGATCGCAAAGAACCAG

© 2020 by the authors. Licensee MDPI, Basel, Switzerland. This article is an open access article distributed under the terms and conditions of the Creative Commons Attribution (CC BY) license (http://creativecommons.org/licenses/by/4.0/).
